# Poor Bifidobacterial Colonization Is Associated with Late Provision of Colostrum and Improved with Probiotic Supplementation in Low Birth Weight Infants

**DOI:** 10.3390/nu11040839

**Published:** 2019-04-13

**Authors:** Katsunori Tanaka, Yoshitaka Nakamura, Masaki Terahara, Takahide Yanagi, Sayuri Nakahara, Ouki Furukawa, Hidemi Tsutsui, Ryo Inoue, Takamitsu Tsukahara, Shigeki Koshida

**Affiliations:** 1 Department of Pediatrics, Shiga University of Medical Science, Seta Tsukinowa-cho, Otsu, Shiga 520-2192, Japan; tanakatsu0124@gmail.com (K.T.); tyanagi@belle.shiga-med.ac.jp (T.Y.); sayuri10@belle.shiga-med.ac.jp (S.N.); ouki@belle.shiga-med.ac.jp (O.F.); hidemi@belle.shiga-med.ac.jp (H.T.); 2 Department of Pediatrics, National Hospital Organization Higashi-Ohmi General Medical Center, Gochi-cho, Higashiomi, Shiga 527-8505, Japan; 3 Food Science & Technology Research Laboratories, Meiji Co., Ltd., Hachiouji, Tokyo 192-0919, Japan; yoshitaka.nakamura@meiji.com (Y.N.); masaki.terahara@meiji.com (M.T.); 4 Department of Agricultural and Life Sciences, Kyoto Prefectural University, Hangi-cho, Shimogamo, Sakyo-ku, Kyoto 606-8522, Japan; r-inoue@kpu.ac.jp; 5 Kyoto Institute of Nutrition & Pathology, Furuikedani, Tachikawa, Ujitawara-cho, Kyoto 610-0231, Japan; tsukahara@kyoto-inp.co.jp; 6 Department of Community Perinatal Medicine, Shiga University of Medical Science, Seta Tsukinowa-cho, Otsu, Shiga 520-2192, Japan

**Keywords:** premature infants, bifidobacteria, colostrum, probiotics

## Abstract

This study aimed to evaluate the association between bifidobacterial colonization in low birth weight infants and perinatal factors, including the timing of initial colostrum and the effect of probiotics on this colonization. In this non-randomized controlled trial, we enrolled 98 low-birth-weight infants from a neonatal intensive care unit (NICU) in Japan. Infants were divided into three groups: group N (no intervention), group H (received non-live bifidobacteria), and group L (received live bifidobacteria). The number of bifidobacteria in the infants’ stools at 1 month of age was measured using real-time polymerase chain reaction (PCR). We divided infants into “rich bifidobacteria” (≥10^4.8^ cells/g feces) and “poor bifidobacteria” (<10^4.8^ cells/g feces) subgroups. The ratio of “rich bifidobacteria” infants was 20/31, 34/36, and 30/30 in groups N, H, and L, respectively. In group N, the “rich bifidobacteria” group received first colostrum significantly earlier than the “poor bifidobacteria” group (1 day vs. 4 days, *P* < 0.05). Compared with the N group, both groups H and L had a significantly high proportion of “rich bifidobacteria” infants (*P* < 0.05). Bifidobacterial colonization was poor in premature infants at 1 month compared with term infants, and the level of colonization was associated with the timing of initial provision of colostrum. Providing probiotics to premature infants can improve bifidobacterial colonization.

## 1. Introduction

Bifidobacteria are commonly found in the intestine of breast-fed term infants; they are initially found in feces on days 2–3 in breast-fed term infants, and then become predominant within the first week of life [1]. Live bifidobacteria provide beneficial effects to host infants (e.g., inhibiting potential pathogenic microorganisms) through secreted substances and/or metabolic products [2]. Their bacterial cell components, such as cell walls, also exert effects on the host, regardless of whether they are alive or dead [3], whereas little has been known about the impact of dead cells on the bifidobacterial colonization.

In premature infants, the intestinal barrier system is immature [4]. The establishment of intestinal flora in premature infants is different from that in term infants not only due to their prematurity, but also due to the unique hospital environment (e.g., cesarean section, antibiotic use, delayed establishment of enteral feeding) [1,5,6,7,8]. Under such conditions, premature infants are born with many risk factors which predispose them to developing necrotizing enterocolitis and sepsis [9,10]. There are reports that abnormal intestinal flora is a risk factor for necrotizing enterocolitis and sepsis in premature infants [11,12]. It has been suggested that the administration of probiotics such as bifidobacteria to premature infants prevents necrotizing enterocolitis and sepsis and shortens the time to establishment of enteral feeding [13,14,15,16]. In addition, in our previous multicenter study [17], oral administration of *Bifidobacterium bifidum* OLB6378 (OLB6378), a strain derived from the intestine of human infants, reduced the incidence of late-onset sepsis in very low birth weight (VLBW) infants.

Bifidobacterial colonization in premature infants is delayed compared with that in term infants [1]. It has been reported that the colonization depends on delivery mode, antibiotic use, and feeding method [2,5,6,8,18,19,20,21,22]. However, the mechanism behind delayed bifidobacterial colonization in premature infants has not been fully elucidated. Thus, to ensure their healthy growth, further investigation of intestinal bifidobacterial colonization in premature infants is important.

Colostrum, the first milk produced by mothers after delivery, contains high concentrations of immunoprotective agents compared with mature human milk [23]. One study showed that administration of colostrum during the first few days of life significantly reduced the occurrence of clinical sepsis with an increase in immunoprotective factors in extremely premature infants [24]. We hypothesized that early provision of colostrum may accelerate the colonization of bifidobacteria, resulting in reducing severe neonatal infection, although the effect of the time of initial provision of colostrum is still poorly understood.

In this study, we evaluated bifidobacterial colonization and related perinatal factors, such as the timing of first colostrum, in low birth weight (LBW) infants at 1 month of age and we examined the influence of the administration of live OLB6378 on the bifidobacterial colonization levels in these infants. In addition, we examined whether partially similar effects would be observed with the administration of non-live OLB6378.

## 2. Materials and Methods

### 2.1. Ethical Statement

This study was registered in the UMIN (University Hospital Medical Information Network in Japan) Clinical Trial Registry (UMIN000020520) and was conducted in accordance with the Declaration of Helsinki. We used fecal samples collected for our previously reported study [25] after obtaining written informed consent from the guardians of the infants according to the approval provided by the Institutional Review Board of Shiga University of Medical Science (approval number 24-151), Japan.

### 2.2. Subjects and Study Protocol

The subject and protocol have been described previously [25], and this study is a secondary analysis focusing on bifidobacterial colonization in LBW infants. The subjects of the study were selected from LBW infants (1500–2500 g) who were admitted in the neonatal intensive care unit (NICU) of Shiga University of Medical Science Hospital between March 2013 and May 2014, and whose legal guardians provided written informed consent for participation in the study within 48 h after birth. We excluded subjects from the study whose legal guardians refused to sign the informed consent form or who had congenital malformations.

We determined the infants to the study groups based on entry order to prevent cross colonization because cross contamination of the control group infants with the probiotic administration to the study group was observed in studies of probiotic use [26]. The subjects were divided into three groups as follows: group N (no intervention), group H (received non-live OLB6378 concentrate), and group L (received live OLB6378 concentrate) in a 1:1:1 proportion, such that each group had >30 subjects. The sample size was determined on the basis of a previous study on the effect of probiotics [18]. We conducted a non-randomized evaluation and made entries in the following order: group H (March 2013 to July 2013), group L (August 2013 to November 2013), and group N (December 2013 to May 2014). The technicians who counted the number of bifidobacteria in the stools of the subjects were blinded to the subjects’ groups, and the legal guardians were not informed of the type of trial compound administered.

### 2.3. Study Intervention

We used a lyophilized probiotic powder containing OLB6378 (Meiji Co., Ltd., Tokyo, Japan), which contains 0.5 g/g of live OLB6378 concentrate, 0.25 g/g of sucrose, and 0.25 g/g of trehalose, as previously reported [25]. For non-live OLB6378, we dissolved live OLB6378 powder in sterile water with a concentration of 10% and lyophilized them by freeze drying after a 10-min heat treatment at 80 °C. The heat-treated batches were subjected to a culture test to ensure that they did not contain any live bacteria.

Group N received no intervention. Group L received a mixture of 20 mg of live OLB6378 powder (containing 10 mg of lyophilized live OLB6378 concentrate with >2.5 × 10^9^ live cells) and 480 mg of dextrin (Matsutani Chemical Industry Co., Ltd., Itami City, Japan), which is the same as the dose used in our previous multicenter study [17]. Group H received a mixture of 20 mg of lyophilized non-live OLB6378 powder (containing 10 mg of lyophilized non-live OLB6378 concentrate with >2.5 × 10^9^ non-live cells) and 480 mg of dextrin. Each mixture (500 mg) was diluted in 1 mL of warm water, mixed with either breast milk or infant formula (depending on the extent of lactation in the mothers), and divided into two 250-mg doses. A 250-mg dose was administered to subjects in each group twice daily. The interventions were started within 48 h after birth and continued for at least 1 month after birth. Prior to discharge from the NICU, the parents of the infants in groups L and H were instructed on how to administer the trial compound to their infants at home and were requested to perform ongoing intervention. Furthermore, after discharge from the NICU, compliance with the use of the trial compound was assessed by interviewing the parents.

### 2.4. Sample Collection and Bacteriological Analyses

For measuring the number of bifidobacteria, the subjects’ stools were collected at 1 month of age. The collected stool samples were immediately frozen at −20 °C. We collected stool samples of the infants on admission in the hospital. In cases of the infants after discharge from the hospital at 1 month of age, we asked the parents to collect their infants’ stool sample and bring them to the hospital at 1 month. There were 14 infants on admission in the hospital and 17 infants were discharged at 1 month of age. Then, bacterial genomic DNA was extracted from stool samples using a commercial extraction kit (QuickGene DNA tissue kit; KURABO, Osaka, Japan) as previously described [27]. Real-time polymerase chain reaction (PCR) was performed with genus-specific primers, which could detect the bifidobacteria genus including the OLB6378 strain. The primer sequences were as follows: Bifidobacterium spp. sense primer, 5′-GATTCTGGCTCAGGATGAACGC-3′; Bifidobacterium spp. antisense primer, 5′-CTGATAGGACGCGACCCCAT-3′ [28]. Each PCR reaction mixture contained 20 pmol of each primer, 5 μL of SYBR^®^ premix Ex taq (Takara Bio, Shiga, Japan), and 1 μL DNA solution in a total volume of 10 μL. The PCR amplification protocol included initial denaturation for 1 min at 95 °C followed by 40 cycles of melting, annealing, and extension at 95 °C for 15 s and 60 °C for 20 s, respectively. The bacterial DNA from stool samples that were too small to be measured were excluded from this analysis.

### 2.5. Characteristics and Feeding Modes

Background information, including gestational age, birth weight, and body weight at 1 month of age, Apgar score, sex, mode of delivery, multiple birth ratio, and antibiotic use during the first month, was collected from the case report forms. The data of initiation time of colostrum intake and breast milk intake rate (breast milk intake/total milk intake) was also collected from the case report forms. We assessed the data that we could confirm exactly when colostrum intake began. As accurate measurements of the feeding methods were not possible during the observation period post-NICU discharge, data were only collected while the infant was in the NICU. We provided formula, not donor milk, to the infants who were unable to obtain their own mother’s milk in the NICU.

### 2.6. Statistical Analysis

Non-normally distributed data were subjected to log transformation before analysis. Differences were evaluated using the Student’s *t*-test, Fisher exact test, or Mann–Whitney *U* test using the Bell Curve for Excel (Social Survey Research Information Co., Ltd. Tokyo, Japan) and by multiple comparisons using Ryan’s method [29]. A *P* value of <0.05 indicated a significant difference.

## 3. Results

### 3.1. Background Characteristics

**Figure 1:**
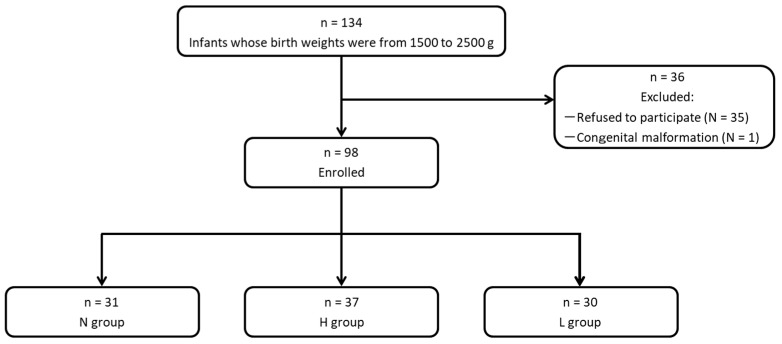
Classification of subjects. N group, non-intervention control; H group, non-live OLB6378; L group, live OLB6378.

As shown in Figure 1, after exclusions, we included 98 infants in the study as follows: group N, *n* = 31; group H; *n* = 37; and group L, *n* = 30, and these infants were evaluated as an intent-to-treat population. As described previously [25], no significant differences were observed in baseline characteristics, such as gestational age, body weight, Apgar score, sex, mode of delivery, or multiple births in the three groups.

### 3.2. Number of Bifidobacteria in the Low Birth Weight (LBW) Infants at 1 Month of Age

**Figure 2:**
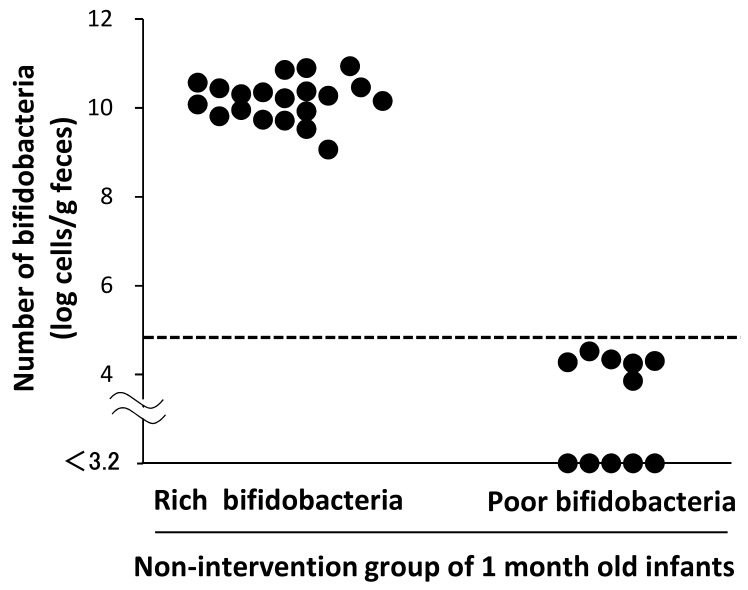
Fecal bifidobacterial counts in the non-intervention control group at 1 month of age. The horizontal dotted line indicates the representative value of the lower limit of the 95% confidence interval of the bifidobacterial counts in Japanese term infants [30].

In the no intervention control group at 1 month of age, who did not receive OLB6378, 20 cases had a bifidobacterial count of >10^8^ cells, and six cases had a count of approximately 10^4^ cells. In five cases, no bifidobacteria were detected. For comparison with the bifidobacterial colonization level in term infants, we set the lower limit of normal bifidobacterial counts in term infants at 10^4.8^ cells/g feces, according to the mean value −2 SD (as the 95% confidence interval) for Japanese term infants in the report by Tsuji et al. [30]. As shown in Figure 2, the 31 infants in the non-intervention control group were divided into a group of 20 cases above the lower limit (the subgroup “rich bifidobacteria”) and a group of 11 cases below the lower limit (the subgroup “poor bifidobacteria”). These subgroups were used for the following comparison analysis.

### 3.3. Comparison of the Characteristics and Feeding History Between the Subgroups

Table 1 shows the characteristics and feeding histories of the subgroups in the no intervention control group. No significant differences in characteristics, including gestational age, birth weight, and body weight at 1 month of age, Apgar score, sex, mode of delivery, multiple birth ratio, and antibiotic use during the first month were found between the subgroups. As feeding history data were only collected for the period when infants were in the NICU, the colostrum start date was confirmed in 30 infants. Regarding the day of initiation of colostrum intake (day, median [interquartile range]), a significant difference was confirmed between the “poor bifidobacteria” subgroup (4 (2–4)) and “rich bifidobacteria” subgroup (1 (1–2)) (*p* = 0.04). An average of breast milk intake rate (breast milk intake/total milk intake) during the observation period was determined. For all periods, the average of breast milk intake rate was lower in the “poor bifidobacteria” subgroup than in the “rich bifidobacteria” subgroup.

### 3.4. Impact of OLB6378 Administration on Fecal Bifidobacterial Counts at 1 Month of Age

**Figure 3:**
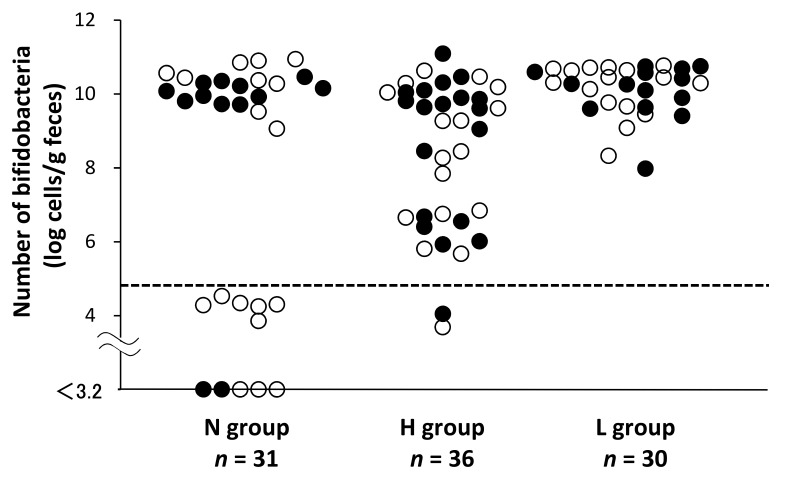
Impact of OLB6378 administration on fecal bifidobacterial counts at 1 month of age. N group, non-intervention control; H group, non-live OLB6378; L group, live OLB6378. The solid dots (●) indicate infants who infants received first colostrum within 48 h of birth, and circles (O) indicate infants that received first colostrum more than 48 h after birth. The horizontal dotted line indicates the representative value of the lower limit of the 95% confidence interval of the bifidobacterial counts in Japanese term infants [30]. Compared with the N group, both the H and L groups had a significantly high proportion of “rich bifidobacteria” infants (*p* < 0.05, by Ryan’s method). One sample in the H group was excluded owing to the lack of microbial DNA from the stool sample.

Figure 3 shows the fecal bifidobacterial counts at 1 month of age for the following three groups: group N, *n* = 31; group H, *n* = 36; and group L, *n* = 30. One sample from the H group was excluded owing to the lack of bacterial DNA from the stool sample. As the probiotic interventions were started within 48 h after the infants’ birth, we divided infants into one group who were first provided colostrum earlier than 48 h after birth and another group who were first provided colostrum later than 48 h after birth. The solid dots (●) indicate infants who ingested colostrum within 48 h of birth, and circles (O) indicate infants that did so after 48 h since birth. We also set the lower limit of normal bifidobacterial counts in term infants at 10^4.8^ cells/g feces. The ratios of infants above the lower limit were 20/31, 34/36, and 30/30, in group N, H, and L, respectively. Furthermore, compared with the N group, both the H and L groups had a significantly high number of infants with bifidobacterial counts above the lower limit set in this study (*P* < 0.05, by Ryan’s method). In addition, the proportion of infants provided colostrum earlier than 48 h among the three groups was not significantly different.

## 4. Discussion

In this study, we found that bifidobacterial colonization in LBW infants at 1 month of age was poor compared with that in term infants, and the level of that colonization was associated with the timing of initial colostrum provision to LBW infants. Next, we found that providing OLB6378 to LBW infants improved the bifidobacterial colonization to the same level as that in the term infants.

First, the bifidobacterial colonization in LBW infants at 1 month was poor compared with that in the term infants in a previous report [30]. In VLBW infants, who are more immature than our study subjects, bifidobacterial colonization is controversial. Sakata et al. [1] reported that bifidobacteria became predominant at approximately 3 weeks after birth; on the other hand, Butcher et al. [31] reported that high levels of bifidobacteria could not be detected even 80 days after birth. In our study, the bifidobacterial counts at 1 month were still lower in more than one-third of premature infants than those in term infants (Figure 2) [30]. Although the reason for the different bifidobacterial colonization compared with that in the previous reports [1,31] is unknown, differences in the hospital environment and infant management are one possible explanation. Importantly, we found that the poor colonization at 1 month was associated with the delayed intake of first colostrum. Previous discussions have identified earlier gestational age [1,8,19], antibiotic administration [8,19,22], and cesarean section [8,19,22] as risk factors for reduced bifidobacterial colonization in premature infants. However, the results of the current study are inconsistent with these findings. Instead, there was a significant association with the timing of the first colostrum feeding. Although it has been reported that the differences in feeding method (breast-fed vs. bottle-fed) is a factor in bifidobacterial colonization in premature infants [21,32], we could not find any study focusing on the implications of the timing of initial colostrum feeding. Thus, to the best of our knowledge, this is the first study to report the association between the timing of initial colostrum feeding and bifidobacterial colonization in premature infants. It is desirable to initiate colostrum feeding in the early postnatal period in LBW infants with birth weight between 1500 and 2500 g to enhance bifidobacterial colonization to the same level as that in term infants. A recent study regarding the association between mode of breast feeding and infantile bacterial colonization indicates that indirect breastfeeding such as pumped milk decreases bifidobacterial colonization [33]. Unfortunately, we were unable to evaluate the mode of breastfeeding. We should consider this effect on bifidobacterial colonization in a future study.

In addition to the poor bifidobacterial colonization in LBW infants, we also found that providing live OLB6378 to LBW infants improved the bifidobacterial colonization to the same level as that in term infants (Figure 3). The current findings are consistent with previous findings wherein bifidobacteria were found to promote bacterial colonization in LBW infants [18,34,35]. Some infants have difficulty receiving colostrum feeding in the early postnatal period due to maternal or infantile problems. Thus, as an alternate strategy in premature infants who cannot receive colostrum, the administration of probiotics such as OLB6378 may confer a health benefit on the infant by enhancing bifidobacterial colonization to the same level as that in term infants.

As shown in Figure 3, a significant increase in bifidobacterial counts was observed after the administration of non-live OLB6378, as well as live OLB6378. It has been shown that live bacterium such as bifidobacteria in the intestine provide beneficial effects to the host through secreted substances and/or metabolic products (e.g., lactic acid, acetic acid, enzymes, and vitamins) [2]. In addition, their bacterial cell components, such as cell walls, also exert effects on the host, regardless of whether they are dead [3]. The cell wall component of Gram-positive bacteria, including bifidobacteria, plays an important role in maintaining health, homeostasis, and mucosal barrier function of the intestinal epithelium and in repairing the intestinal epithelium [36,37,38]. Therefore, in order to maintain the health of the intestinal epithelium, it is considered meaningful to influence the composition of bacterial cell components in the intestine, even if they are dead. For infants who are born with LBW and are bottle-fed in particular, administering sterilized cell components of bifidobacteria is considered to be beneficial in altering the composition of bacterial cell components in the intestine, similar to that in full-term and breast-fed infants.

There were some limitations in our study. First, the data on bifidobacterial colonization in term infants were quoted data that were not measured under the same conditions as those in this study. Thus, the results of the comparison between LBW infants and term infants may be inaccurate. Second, this study could not employ multiple regression analysis to identify other confounding factors due to the small sample size. However, there have been no reports showing that the timing of initiation of colostrum feeding affects bifidobacterial colonization in LBW infants. Thus, further investigation regarding the effects of the timing of initial colostrum feeding on bifidobacterial colonization may contribute to the understanding of the intestinal flora in premature infants. Additionally, as we had not given dextrin as placebo to infants in N group, we could not conclude that dextrin had any effects on bifidobacterial colonization.

## 5. Conclusions

We showed that bifidobacterial colonization of LBW infants at 1 month was poor compared with that of the term infants, and the level of that colonization was associated with the timing of initial colostrum in LBW infants. We also showed that providing OLB6378 to LBW infants increased the bifidobacterial colonization to the same level as that in term infants. Further studies on bifidobacterial colonization in premature infants are required for the neonatal healthy growth during hospitalization in the NICU.

## Figures and Tables

**Table 1:** Comparison of characteristics and feeding histories of subjects between the subpopulation in the N group.

	Poor Bifidobacteria	Rich Bifidobacteria	
	*n* = 11	*n* = 20	*P* Value
Characteristics			
Gestational age, weeks ^a^	36.1 ± 1.9	35.1 ± 1.6	0.11 †
Body weight at birth, g ^a^	1930 ± 275	2081 ± 234	0.13 †
Body weight at 1 month, g ^a^	2481 ± 315	2808 ± 552	0.09 †
Apgar score at 1 min ≤ 3 ^b^	0 (0)	0 (0)	1.00 ‡
Apgar score at 5 min ≥ 7 ^b^	11 (100)	20 (100)	1.00 ‡
Male sex ^b^	4 (36)	10 (50)	0.36 ‡
Cesarean section ^b^	9 (82)	16 (80)	0.65 ‡
Multiple births ^b^	7 (64)	7 (35)	0.12 ‡
Antibiotic administration during the first month ^b^	0 (0)	3 (15)	0.25 ‡
Time of initiation of colostrum intake, day ^c,d^	4 (2–4) [*n* = 11]	1 (1–2) [*n* = 19]	0.04 §
Average of breast milk intake rate (breast milk intake/total milk intake)			
during 0–7 day ^c^	0.08 (0.01–0.22)	0.24 (0.13–0.33)	0.04 §
during 8–14 day ^c,d^	0.63 (0.33–0.80) [*n* = 8]	0.73 (0.63–0.88) [*n* = 16]	0.27 §
during 15–21 day ^c,d^	0.56 (0.19–0.89) [*n* = 8]	1.00 (0.63–1.00) [*n* = 12]	0.04 §
during 22–28 day ^c,d^	0.30 (0.09–0.63) [*n* = 6]	0.89 (0.47–1.00) [*n* = 8]	0.052 §

N group, no intervention control, ^a^ Mean ± standard deviation, ^b^ Number (%), ^c^ Median (interquartile range), ^d^ Number of infants acquired feeding records during NICU admission. † Student’s *t*-test, ‡ Fisher exact test, § Mann–Whitney *U* test, *P* values of <0.05 are given in bold.
